# Application of Differential Evolution Algorithm on Self-Potential Data

**DOI:** 10.1371/journal.pone.0051199

**Published:** 2012-12-11

**Authors:** Xiangtao Li, Minghao Yin

**Affiliations:** 1 Faculty of Chemistry, Northeast Normal University, Changchun, People's Republic of China; 2 College of Computer Science, Northeast Normal University, Changchun, People's Republic of China; Université de Nantes, France

## Abstract

Differential evolution (DE) is a population based evolutionary algorithm widely used for solving multidimensional global optimization problems over continuous spaces, and has been successfully used to solve several kinds of problems. In this paper, differential evolution is used for quantitative interpretation of self-potential data in geophysics. Six parameters are estimated including the electrical dipole moment, the depth of the source, the distance from the origin, the polarization angle and the regional coefficients. This study considers three kinds of data from Turkey: noise-free data, contaminated synthetic data, and Field example. The differential evolution and the corresponding model parameters are constructed as regards the number of the generations. Then, we show the vibration of the parameters at the vicinity of the low misfit area. Moreover, we show how the frequency distribution of each parameter is related to the number of the DE iteration. Experimental results show the DE can be used for solving the quantitative interpretation of self-potential data efficiently compared with previous methods.

## Introduction

The self-potential (SP) method is one of the most important optimization problems in geophysics, which is on the basis of the measurement of natural occurring potential difference generated chief from electrochemical, electro kinetic and thermoelectric sources. The SP method has been used in a wide range of applications in geophysics, including prospecting purposes [1], [2], archaeology [3], engineering problem [4], cave detection [5], [6] and geothermal exploration [7], [8]. In some conditions, the SP anomaly can be defined by a single polarize body assuming a model of simple geometry like a sphere, a horizontal or vertical cylinder or an inclined sheet. During the past decade, we have viewed different kinds of methods advanced to handle the SP problem: the method of characteristic points [9]; the curve matching method [10], [11]; the method of least square approaches [12], [13]; the method of derivative analysis and gradients [14]. On the other hand, other techniques have also been used on such as a numerical gradient method, direct interpretation. These techniques can be categorized into two parts: derivative-based method and global search method.

Recently, the differential evolution algorithm [15] has been proposed as a simple and powerful population-based stochastic optimization, which is originally motivated by the mechanism of natural selection. This algorithm searches solutions using three basic operators: mutation, crossover and greedy selection. Mutation is used to generate a mutant vector by adding differential vectors obtained from the difference of several randomly chosen parameter vectors to the parent vector. After that, crossover operator generates the trial vector by combining the parameters of the mutated vector with the parameters of a parent vector selected from the population. Finally, according to the fitness value, selection determines which of the vectors should be chosen for the next generation by implementing one-to-one completion between the generated trail vectors and the corresponding parent vectors. In order to accelerate the convergence speed and avoid the local optima, several variations of DE have been proposed to enhance the performance of the standard DE recently. Moreover, DE has been proved to be really efficient when solving real world problems [16]–[23].

In this paper, we will use the differential evolution to perform the SP method for mineral exploration to find some model parameters. In this study, SP data can be interpreted as a simple geometrical body approximation including a sphere, cylinder, dyke and so on. Then we use our algorithm to estimate the SP data such as Süleymanköy anomaly, Weiss anomaly and Nalbant cesmesi anomaly. In order to demonstrate the advantages of the proposed design, the results obtained are compared with other state-of-the-art approaches. The experimental results show that the DE algorithm is very competitive.

## Materials and Methods

### 0.1 Formulation of the Problem

Generally speaking, the inverse problem can be described as follows:
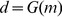
(1)where d represents a set of observations and m denotes a set of model parameters, and G is a non-linear forward operator. The main purpose of the process is estimated between observed data and correct parameters of a chosen model. Eq.(1) is ill posed, because of the forward operator is non-linear one. Eq. (1) can also be defined as follows:
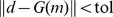
(2)where tol is a predetermined threshold value such as 0.001. Eq (2) is effective for any sampling problem.

**Figure 1:**
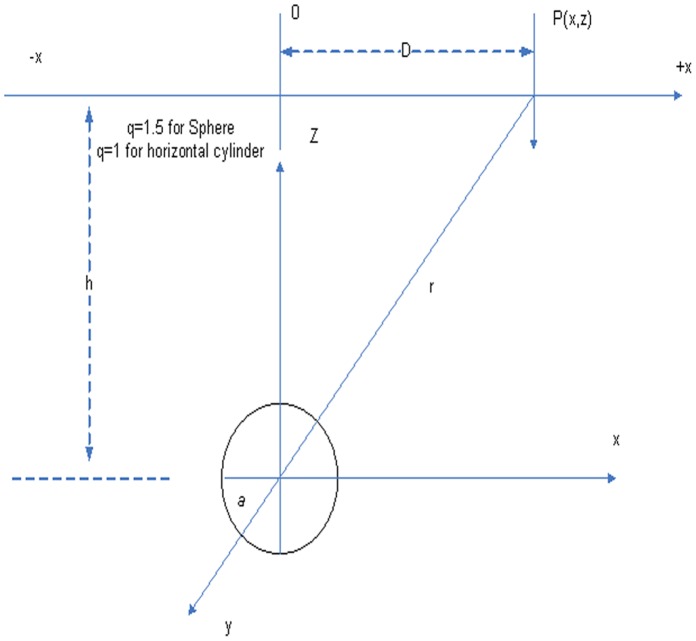
Two simple geometrical bodies under the surface.

The SP anomaly at any point P(x) over a sphere or a cylinder-like body can be described in Figure 1. Figure 1 shows the geometry of a sphere and horizontal cylinder in a medium. The following denotation can be used [24].
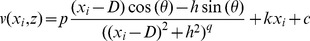
(3)where *P* is electrical dipole moment, 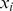 is the coordinate of an estimation point at the outside along the profile. 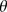 is the polarization angle and h is the deepness from the beginning. *k* is the electric current dipole moment, which denotes the base slope and C represents the base level. The shape factors *q* is 1.5 and 1.0 for a sphere and cylinder, respectively. In section 4, we will investigate to find model parameters of synthetic data and noise added data by using the differential evolution algorithm.

### 0.2 Differential Evolution Algorithm

Differential Evolution (DE) [15] is a population based stochastic search algorithm for solving optimization problems in continuous space. Different from other algorithms, DE uses the distance and direction information from current population to guide its further search. The fundamental crucial idea behind DE is a scheme for producing trial vectors according to the manipulation of target vector and difference vector. If the trail vector yields a lower objective function than a predetermined population member, the newly generated trail vector will replace the vector and be compared in the following generation.

The algorithm begins with a randomly initiated population which utilize NP D-dimension parameter vector within constrained by the prescribed minimum and maximum bounds.
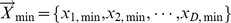


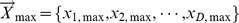



Therefore, we may generate the *j*th component of the *i*th vector as.
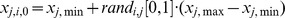



Where 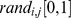 is a uniformly distribution random number between 0 and 1. *i* = 1, …, NP and *j* = 1,…,D.

After initialization, mutation vectors 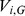 are generated according to each population member or target vector 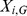 in current population. In the standard DE algorithm, five differential mutation strategies can be used with one of two different crossover methods. There are listed in the following:

“DE/rand/1”.
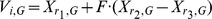



“DE/best/1”.
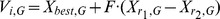



“DE/current-to-best/1”.
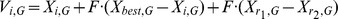



“DE/best/2”.
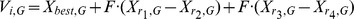



“DE/rand/2”.
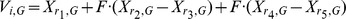



Where 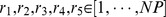 are randomly chosen integers, and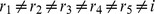. F is the scaling factor controlling the amplification of the differential evolution. 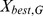 is the best individual vector with the best value in the population at generation *G*.

In the crossover operation, a recombination of the donor vector 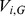 and the parent vector 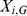 produce a trail vector 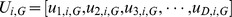. Basic DE employs the binomial crossover defined as follows:
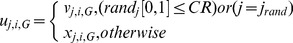
(4)


where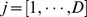;
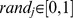
is a uniformly distribution distributed random number; 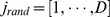 is the randomly chosen index, CR is the crossover rate 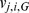 is the difference vector of the *j*th particle in the *i*th dimension at the *G*th iteration, and 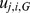 denotes the trail vector of the *j*th particle in the *i*th dimension at the *G*th iteration.

Selection operator is used to choose the next population (i.e. G = G+1) between the trail population and the target population. The selection operation is described as:



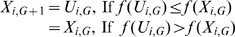
(5)




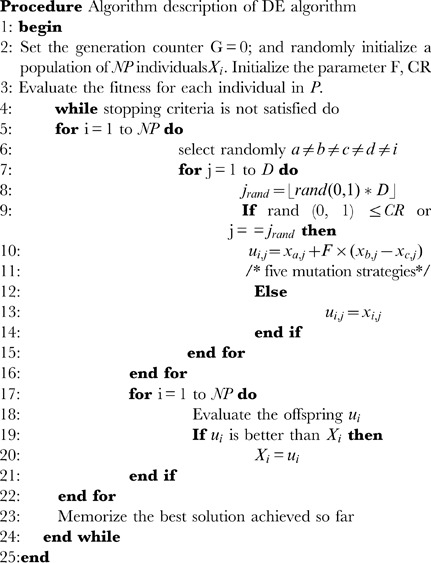



In this work, the objective function (Relative Error) can be calculated as follows:

Relative Error = 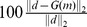


## Results

In this paper, noise-free, contaminated and field data are used to demonstrate the performance and applicability of the proposed differential evolution and particle swarm optimization algorithm. The software is written in Matlab-7 language and experiments are made on a Pentium 3.0 GHz Processor with 1.0 GB of memory. In the parameter of PSO [25]: population size is 300. The number of generation is 100. *c_1_* and *c_2_* are set to be 1.8 and 2.0, respectively. And *v_max_* is 5% of the search space. The inertia weight is set to 0.8. The following differential evolution parameters have been used after a number of careful experimentation: The population size is 300, the number of generation is 100, the scale factor F and crossover probability CR is 0.5, and 0.9, respectively. For the five mutation strategies of DE, “DE/current-to-best/1” was used in our experiment.

Parameter ranges or the dimension of search space used here must be determined before the differential evolution process. The dimensions of the following ranges are used for the DE similar to the PSO: such as P being between −10,000 and 100,000, D being between 1 and maximum of x (the distance), h being between 0 and 500, 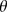 set to −180 and 180°, M set to −20 and 20, and as for the last parameter C set to −1000 and 1000.

### 0.3 Noise-free Data

**Figure 2:**
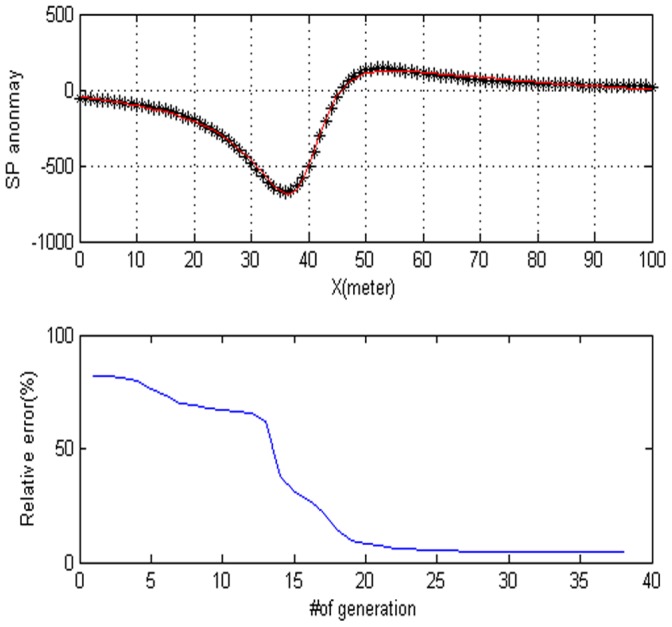
Noise-free data for a cylinder. (a) Dotted lines synthetic data for a cylinder. Estimated SP anomaly is illustrated by solid line. (b) The convergence rate of the DE algorithm.

In this section, we will use the differential evolution for estimate a simple cylinder. The parameters of the model are polarization angle, the depth of the body center, the distance from the origin, the base slope, and the based level. First, we demonstrate some results with the synthetic data set for a cylinder model. The experimental results of PSO and DE are shown in table 1. If the objective value if lower than the threshold value with 0.1% on the two iterations, then we think it is success. In table 1, the true values represent the correct values of parameters with the purpose of comparison. The estimated values are given in third column. The successes rate, mean iteration, std. iterations, mean function evaluation, std. function evaluation, std. RMS, mean RMS and Min RMS of PSO and DE are listed in column four and column seven. Compared with the PSO algorithm, we find that the DE algorithm can provide smaller RMS (0.001578) than PSO algorithm with little iterations. For the success rate, DE algorithm can obtain all conditions. The anomaly of the corresponding model is shown in Figure 2. Figure 2(a) shows the model response. Figure 2(b) shows the fitness behavior. From figure 2(b), after 50 generations, there are no significant changes.

**Table 1:** Comparison of true and estimated parameters of SP anomaly.

		PSO			DE		
Parameter	True	Estimated	Statistics of PSO		Estimated	Statistics of DE	
Elect.dip.mom.	100,000	99,9994.0000	Successes	26/30	100,000	Successes	**30/30**
Distance	40	40.0001	Mean iter.	59.69	40	Mean iter.	51.70
Depth	10	9.9950	Std. iterations	27.22	10	Std. iterations	14.23
Pol. angle(degree)	60	60.0050	Mean func. eval.	18207.69	60	Mean func. eval.	15810.00
Base slope	0	0.0011	Std func. eval.	8166.95	5.2255e–005	Std func. eval.	4270.46
Base level	0	−0.1102	Std RMS	3.20	−0.0017318	Std RMS	1.10
			Mean RMS	1.73		Mean RMS	0.42
			Min RMS	0.02697		Min RMS	**0.0015785831**

**Figure 3:**
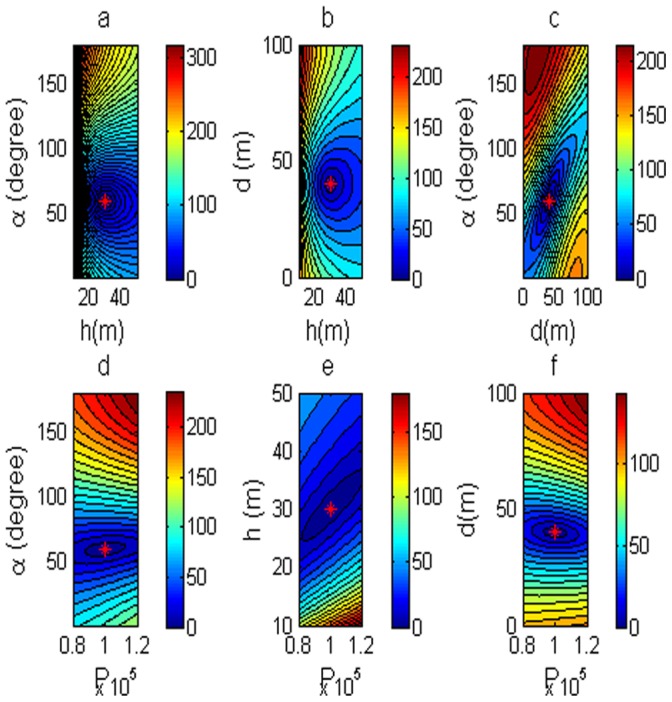
Contour maps of low misfit areas with noise free data. Two of six parameters are variable, rest of them are fixed.

**Figure 4:**
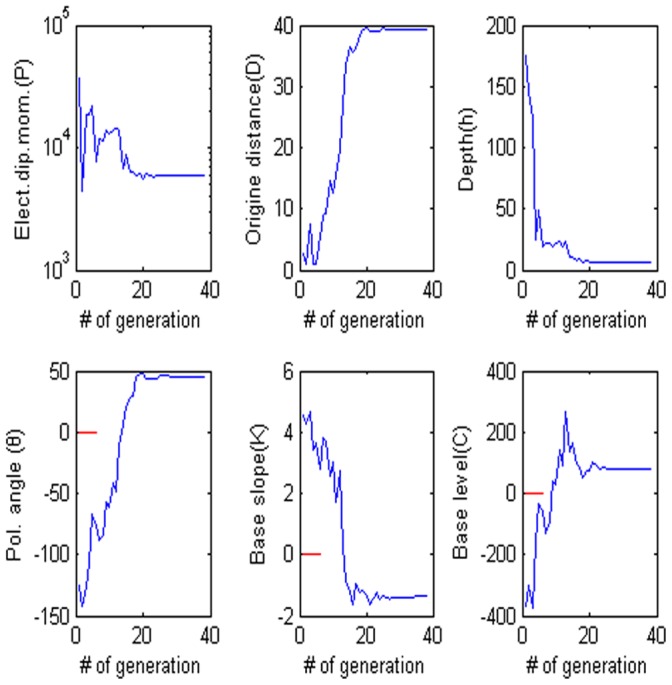
Values of each parameter versus the number of generation. (a) Amplitude P, (b) the distance from the origin D, (c) depth h, (d) polarization angle, (e) base slope K and (f) based level C.

**Figure 5:**
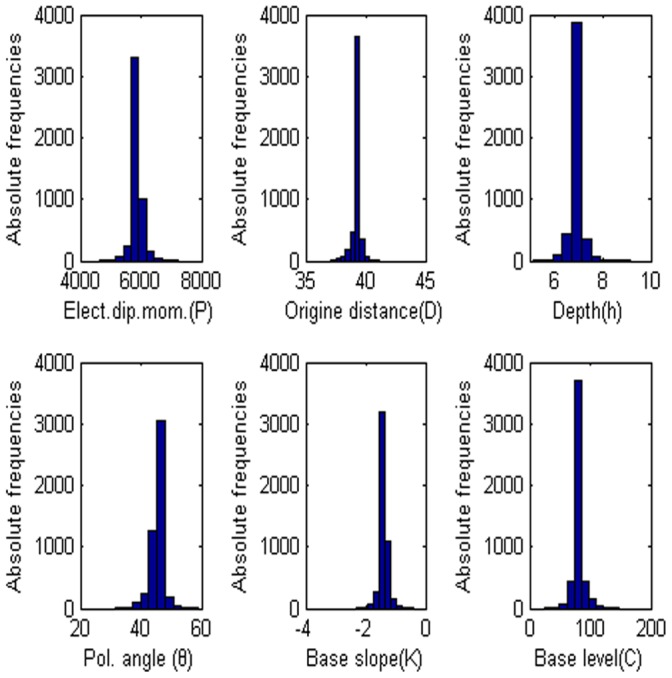
Frequency distribution of each parameter. (a) Amplitude P, (b) the distance from the origin D, (c) depth h, (d) polarization angle, (e) base slope K and (f) based level C.

The inverse problem has 6 unknown parameters need to be solved. The problem is a simple 2-dimension problem. In order to dissent the performance of errors for estimating the model parameter by using the DE, Figure 3 shows the polarization angle and the depth of the body center as variables. Then, two of six parameters are variables, and the rest are fixed as P = 100,000, D = 40 m, h = 30 m, 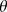 = 60°, M = 0, and C = 0. The low misfit area and the exact point on the map use a star to express it. The rest of the panels are also shown in Figure 3. Figure 4(a–f) shows the behavior of the corresponding parameters. Figure 5 shows high frequencies with the respect to the correct value of parameters.

### 0.4 Contaminated Synthetic Data

**Figure 6:**
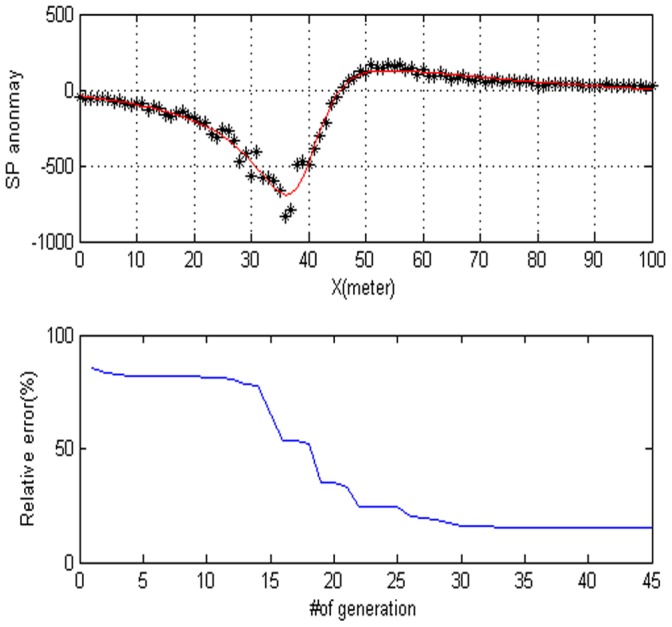
Contaminated synthetic data with 50% Gaussian noise for a cylinder. (a) Dotted lines synthetic data for a cylinder. Estimated SP anomaly is illustrated by solid line. (b) the convergence rate of the DE algorithm.

**Figure 7:**
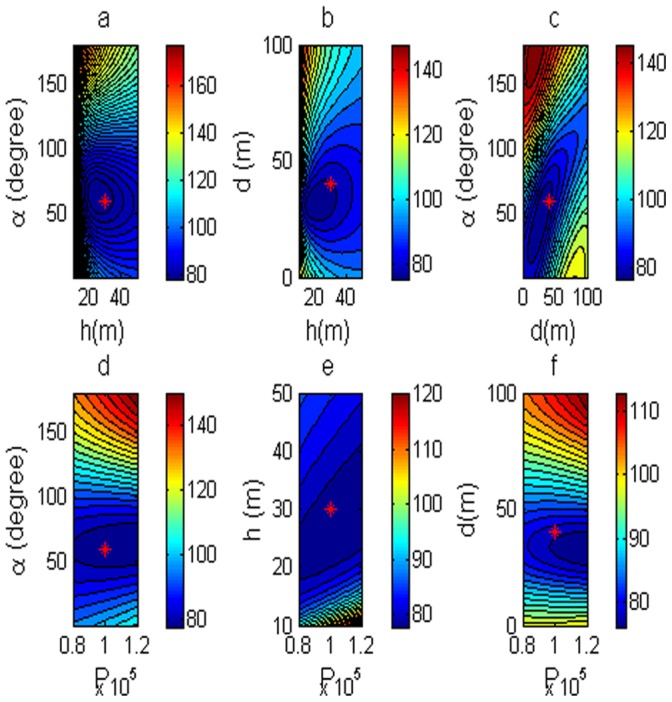
Contour maps of low misfit areas with noise free data. Two of six parameters are variable, rest of them are fixed.

**Figure 8:**
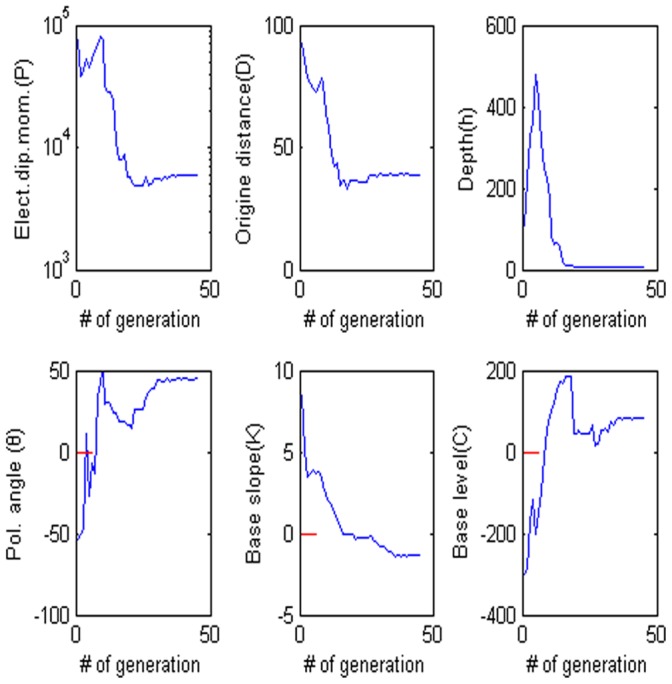
Values of each parameter versus the number of generation. (a) Amplitude P, (b) the distance from the origin D, (c) depth h, (d) polarization angle, (e) base slope K and (f) based level C.

**Figure 9:**
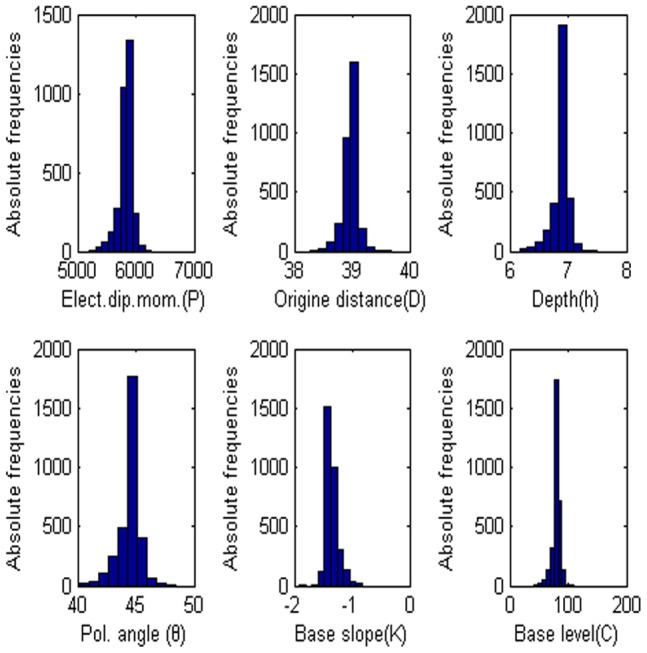
Frequency distribution of each parameter. (a) Amplitude P, (b) the distance from the origin D, (c) depth h, (d) polarization angle, (e) base slope K and (f) based level C.

Noisy data plays an important role in both geophysics and other sciences as well. In order to analyze the behavior of noise corrupted data, we will add some noise to the data set. Figure 6 shows the anomaly of the corresponding model. The parameter obtained from the DE inversions considering noise free data with Gaussian noise added on the values are shown in Table 2. Figure 7 shows the contour maps that illustrate the low misfit area and the exact point the map with a star. Figure 8 shows the behavior of the corresponding parameters. According to the frequency distribution, the mode of the histogram provided the correct parameter values. Figure 9 show the high frequency with the respect to the correct value of parameters. It can be noted that the parameters are quite well recovered from the DE inversion.

**Table 2:** Comparison of true and estimated parameters of SP anomaly with 50% Gaussian noise caused by a cylinder model.

		DE		
Parameter	True	Estimated	Statistics of DE	
Elect.dip.mom.	100,000	102700	Successes	30/30
Distance	40	39.679	Mean iter.	36.43
Depth	10	10.06	Std. iterations	7.19
Pol. angle(degree)	60	59.186	Mean func. eval.	11230.00
Base slope	0	−0.10591	Std func. eval.	2157.13
Base level	0	9.5708	Std RMS	1.09
			Mean RMS	13.54
			Min RMS	13.2577

### 0.5 Süleymanköy Anomaly

**Figure 10:**
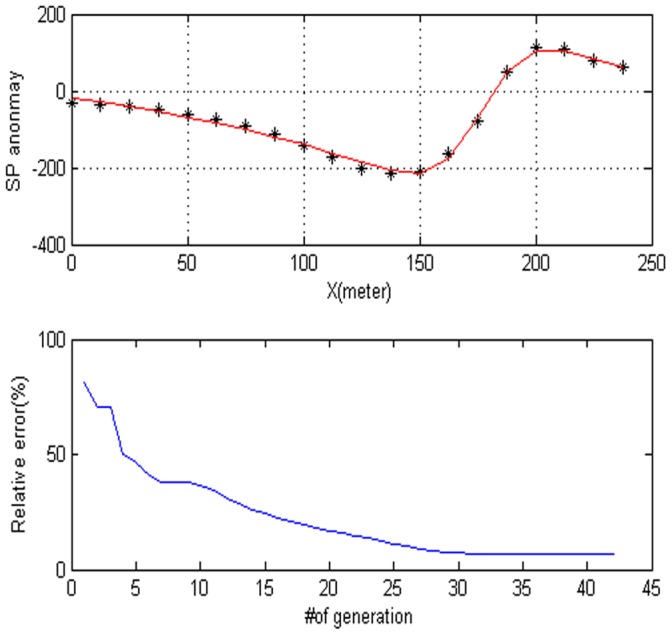
The data used here are digitized from Yungul (1950) algong A–M profile, it is called suleymankoy anomaly.

**Figure 11:**
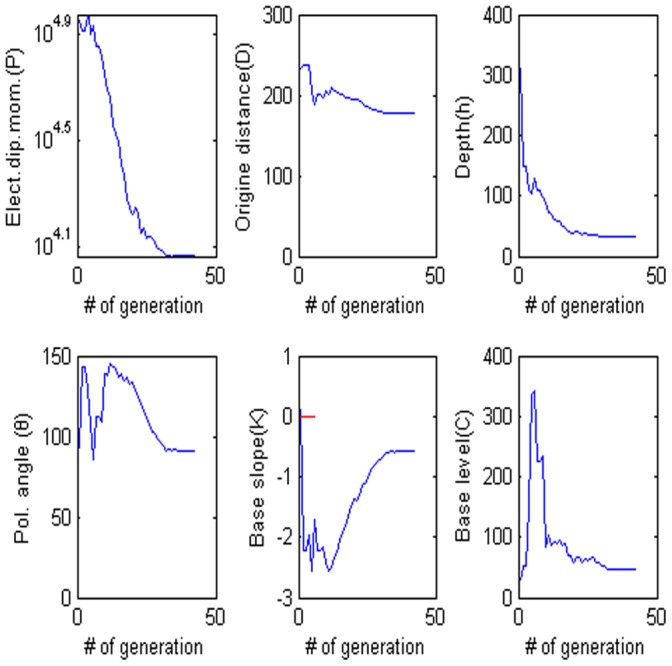
Values of each parameter versus the number of generation. (a) Amplitude P, (b) the distance from the origin D, (c) depth h, (d) polarization angle, (e) base slope K and (f) based level C.

**Figure 12:**
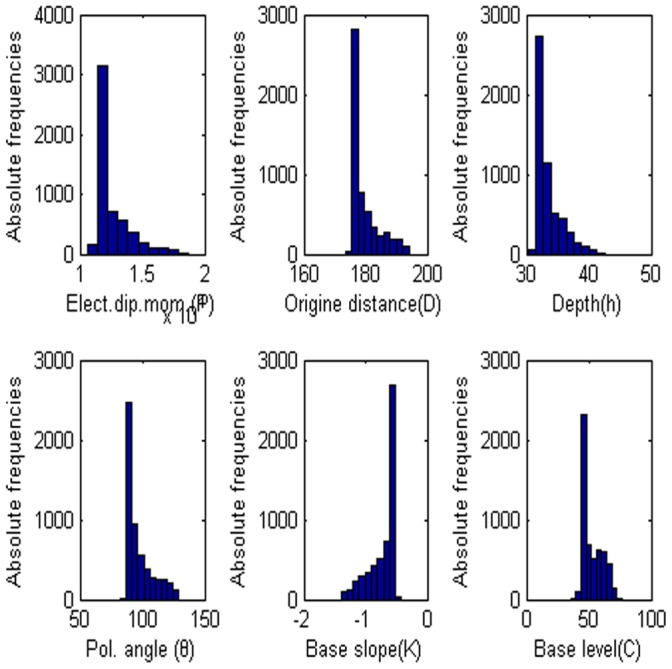
Frequency distribution of each parameter. (a) Amplitude P, (b) the distance from the origin D, (c) depth h, (d) polarization angle, (e) base slope K and (f) based level C.

Süleymanköy anomaly is a quite well-known data set in the literature. The length of profile is 237.5 m. Data are digitized at intervals of 12.5 m along the profile. The algorithm has been executed for 30 times with differential initial populations and the experimental result as the final optimization solution has been selected. The experimental results obtained from the DE are compared with those obtained by using other algorithms including Yungul (1950), Bhattacharya and Roy (1981), Rarm Babu and Rao (1981), Abdelrahman and sharafeldin (1997), Abedlrahman et al.(1997a), El-Araby (2004), Peksen et al (2010) [Bibr pone.0051199-ElAraby1] [Bibr pone.0051199-Rao1] [Bibr pone.0051199-Peksen1] [Bibr pone.0051199-Yngl1] [Bibr pone.0051199-Bhattacharya1] [Bibr pone.0051199-Abdelrahman3] [13,24–29] in terms of different parameter, in Table 3, which shows that DE successes in finding the best solutions for the test methods. Compared with the PSO algorithm, the DE algorithm can provide better solutions than PSO algorithm. The DE can provide 28 successes rate compared with the 14 times of PSO. For the DE algorithm, the parameters are set as follows: the polarization angle 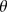 = −90.688, the electrical dipole moment is P = 11,757 mV, the depth of the body center is 32.661, the distance is 176.73 m and the best fitness 6.55. The DE algorithm and the Süleymanköy anomaly are given in Figure 10(a). The convergence rate for the proposed algorithm for the corresponding anomaly is depicted in Figure 10(b). In this model, we use the sphere model. Figure 11 shows the behavior of each parameter. Figure 12 shows the frequency distribution of each parameter by using Süleymanköy anomaly.

**Table 3:** PSO results and statistics of suleymankoy anomaly.

Parameter	DE	PSO	Yungul	Bhattacharya and Roy	Abdelrhman and Sharafeldin	EI-Araby	Abdelrhman	Ram Babu and Rao
Elect.dip.mom.	11757	11704	–	–	−2458	−2661.2	−1549.3	–
Distance	76.73	77.21	76.70	70.00	–	–	–	66.36
Depth	32.661	32.501	38.00	40.00	42.00	47.63	38.78	41.40
Pol. angle(degree)	90.688	91.651	−69.00	−75.00	−77.00	−75.26	−75.33	−86.92
Base slope	−0.57907	−0.58242	0.00	0.00	–	–	–	0.22
Base level	47.016	45.559	0.00	0.00	–	–	–	43.00
PSO					DE			
Success	14/30	Std func. eval.	9984.21		Success	28/30	Std func. eval.	3647.83
Mean iter	62.71	Std RMS	12.19		Mean iter	43.00	Std RMS	9.58
Std. iterations	33.28	Mean RMS	16.85		Std. iterations	12.16	Mean RMS	14.65
Mean func. eval.	19114.29	Min RMS	6.5654		Mean func. eval.	13200.00	Min RMS	**6.546**

### 0.6 Weiss Anomaly

**Figure 13:**
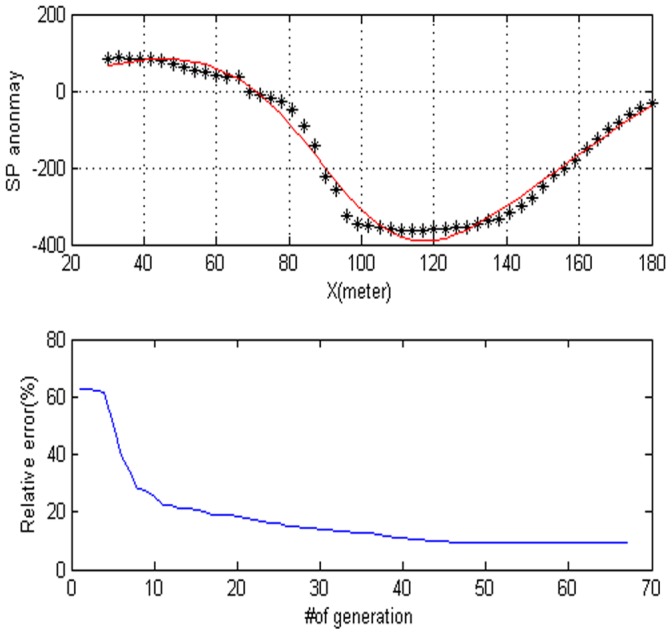
The data used here are digitized from Yungul (1950) algong A–M profile, it is called weiss anomaly.

**Figure 14:**
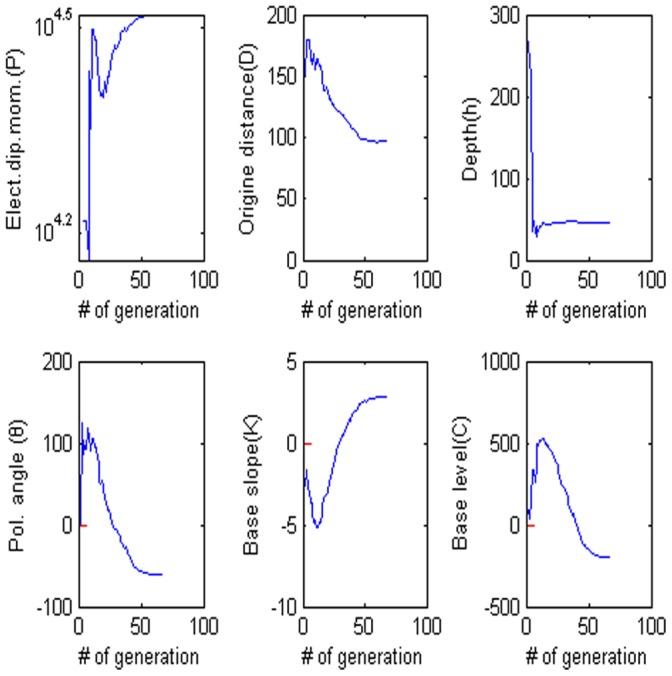
Values of each parameter versus the number of generation. (a) Amplitude P, (b) the distance from the origin D, (c) depth h, (d) polarization angle, (e) base slope K and (f) based level C.

**Figure 15:**
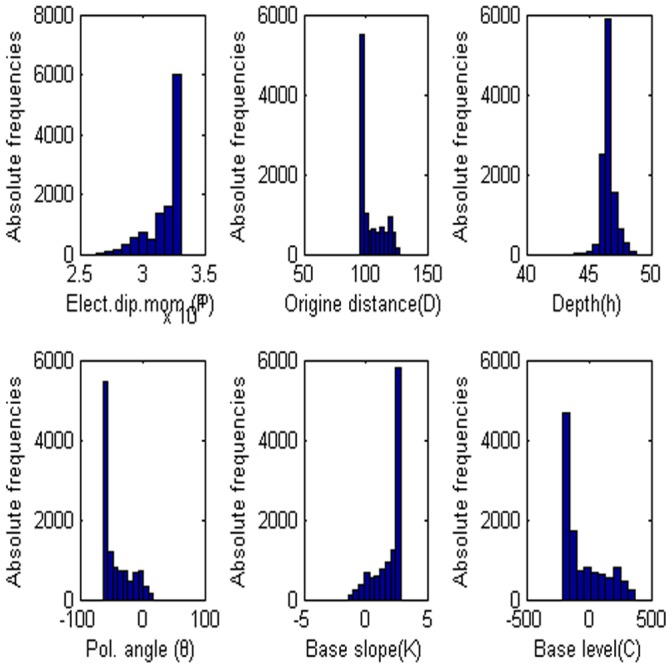
Frequency distribution of each parameter. (a) Amplitude P, (b) the distance from the origin D, (c) depth h, (d) polarization angle, (e) base slope K and (f) based level C.

Weiss anomaly is a quiet well-known data set in the literature. The algorithm has been executed for 30 times with differential initial populations and the experimental result as the final optimization solution has been selected. The experimental results obtained from the DE are compared with those obtained by using other algorithms including Yungul (1950), Bhattacharya and Roy (1981), Rarm Babu and Rao (1988), Abdelrahman and sharafeldin (1997), El-Araby (2004), Peksen et al (2010) [Bibr pone.0051199-ElAraby1] [Bibr pone.0051199-Peksen1] [Bibr pone.0051199-Yngl1] [Bibr pone.0051199-Bhattacharya1] [13,25–28] in terms of different parameter, in Table 4, which shows that DE can beat other algorithms. Compared with the PSO algorithm, the DE algorithm can provide better solution than PSO algorithm. The DE can provide 29 successes rate than the 27 times of PSO. For the DE algorithm, the parameters set as follows: the polarization angle 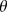 = −62.102, the electrical dipole moment is P = −35,406 mV, the depth of the body center is 47.883, the distance is 96.155 m and the best fitness 9.36 better than the best fitness 9.83 of PSO algorithm. Figure 13a and b show digitized the DE algorithm and the Weiss anomaly and the convergence rate for the proposed algorithm for the corresponding anomaly, respectively. In this model, we also use the sphere model. Figure 14 shows the behavior of each parameter. Figure 15 shows the frequency distribution of each parameter by using Weiss anomaly.

**Table 4:** DE results and statistics of Weiss anomaly.

Parameter	DE	PSO	Yungul	Bhattacharya and Roy	Abdelrhman and Sharafeldin	EI-Araby	Ram Babu and Rao
Elect.dip.mom.	−35406	−29461	–	–	–	–	−13253.80
Distance	96.155	101.57	105.70	108.20	–	–	109.83
Depth	47.883	44.453	53.80	54.00	52.90	43.37	61.05
Pol. angle(degree)	−62.102	−47.681	−64.00	−60.00	−54.70	−54.11	−56.17
Base slope	3.0699	1.9401	0.00	0.00	–	–	1.23
Base level	−219.14	−72.32	0.00	0.00	–	–	−5.48
PSO				DE			
Success	27/30	Std func. eval.	6397.01	Success	29/30	Std func. eval.	7679.60
Mean iter	48.07	Std RMS	1.63	Mean iter	40.43	Std RMS	8.15
Std. iterations	21.32	Mean RMS	11.58	Std. iterations	25.60	Mean RMS	14.99
Mean func. eval.	14722.22	Min RMS	9.8338	Mean func. eval.	12428.57	Min RMS	**9.3594**

### 0.7 Nalbant Cesmesi Anomaly

**Figure 16:**
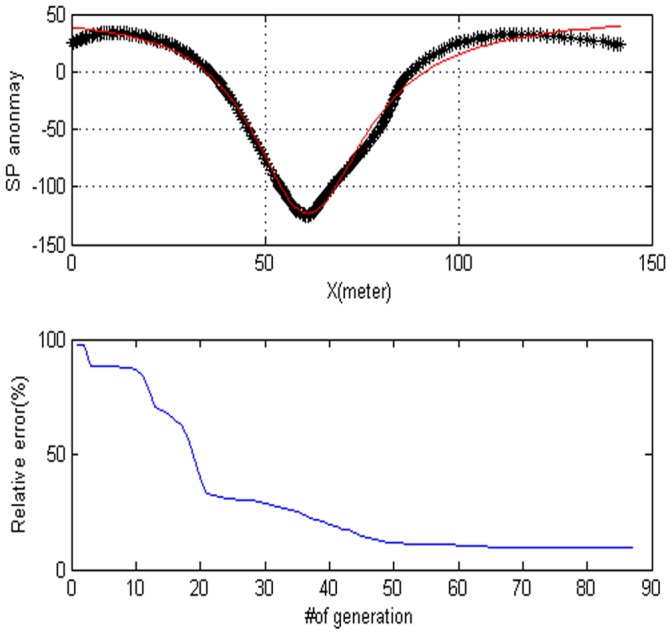
The data used here are digitized from Yungul (1950) algong A–A’ profile, it is called Nalbant cesmesi anomaly.

**Figure 17:**
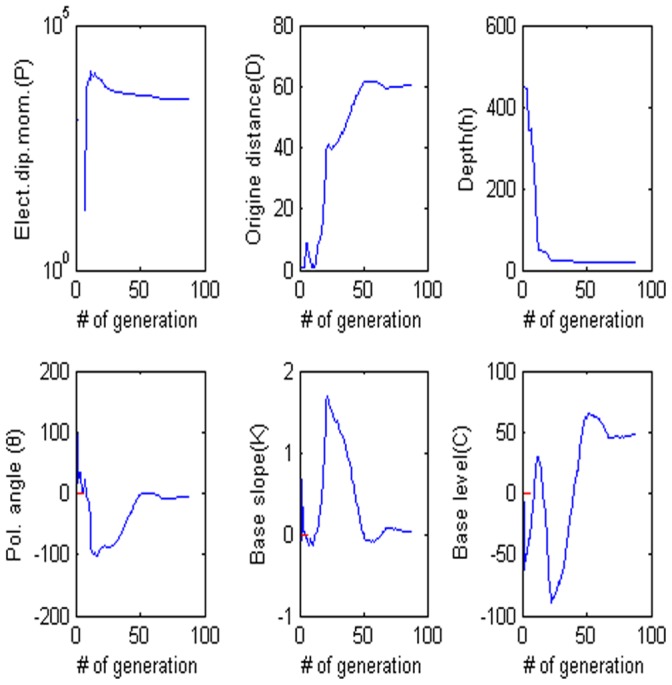
Values of each parameter versus the number of generation. (a) Amplitude P, (b) the distance from the origin D, (c) depth h, (d) polarization angle, (e) base slope K and (f) based level C.

**Figure 18:**
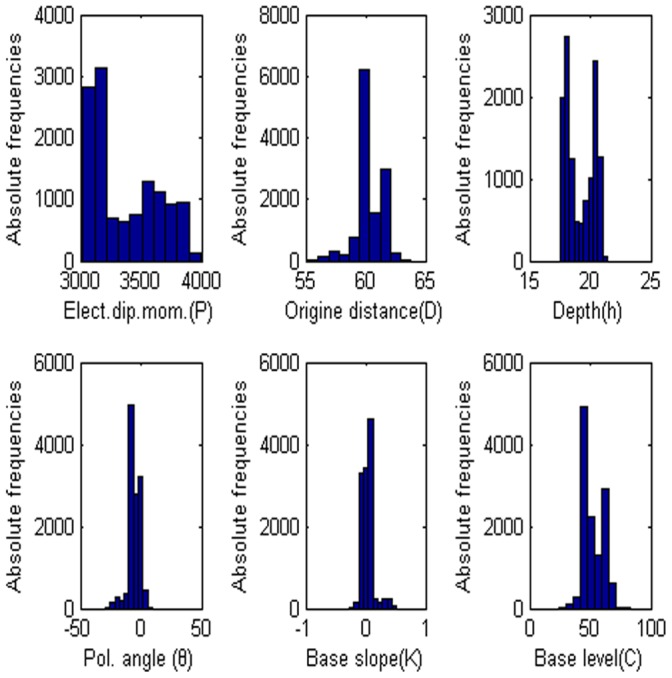
Frequency distribution of each parameter. (a) Amplitude P, (b) the distance from the origin D, (c) depth h, (d) polarization angle, (e) base slope K and (f) based level C.

In this section, we will use the DE algorithm to estimate the parameter of Nalbant cesmesi anomaly. The algorithm has been executed for 30 times with differential initial populations and the experimental results as the final optimization solution have been selected. The experimental results obtained from the DE are compared with those obtained by using other algorithms including Yungul (1954), Abedelrahman et al (2008), Peksen et al (2010) [Bibr pone.0051199-Peksen1] [Bibr pone.0051199-Yngl2] [25,30–31] in terms of different parameters, in Table 5, which shows that DE can beat other algorithms. In this model, we also use the horizontal cylinder model. Compared with the PSO algorithm, the DE algorithm can provide better solution than PSO algorithm. The DE can provide 22 successes rate than the 14 times of PSO. For the DE algorithm, the parameters are set as follows: the polarization angle 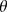 = −8.5744, the electrical dipole moment is P = 84597 mV, the depth of the body center is 22.699, the distance is 60.121 m and the best fitness is 8.08 better than the best fitness 8.10 of PSO algorithm. Figure 16(a) shows that anomaly along the profile and Figure 16(b) shows the fitness value versus the number of generation. Figure 17 shows the behavior of each parameter. Figure 18 shows the frequency distribution of each parameter by using Nalbant cesmesi anomaly.

**Table 5:** DE results and statistics of Nalbant cesmesi anomaly.

				PSO		DE	
Parameter	DE	PSO	Abdelrhman et al	Estimated	Statistics of PSO	Estimated	Statistics of DE
Elect.dip.mom.	84806	84597	3403.12	Successes	14/30	Successes	22/30
Distance	59.754	60.121	–	Mean iter.	66.64	Mean iter.	45.86
Depth	22.702	22.699	26.23	Std. iterations	21.24	Std. iterations	16.87
Pol. angle(degree)	−10.401	−8.5744	−68.53	Mean func. eval.	20292.86	Mean func. eval.	14059.09
Base slope	0.017072	0.014053	–	Std func. eval.	6371.14	Std func. eval.	5061.87
Base level	40.042	40.492	–	Std RMS	4.50	Std RMS	6.36
				Mean RMS	10.19	Mean RMS	9.85
				Min RMS	8.1106182767	Min RMS	**8.0862**

## Discussion

### 0.8 Compared with Other State of the Art DE Algorithms

In order to evaluate the effectiveness and efficiency of our method, we further compare its performance with some state-of-art DE algorithms. They are jDE [16], DE/BBO [19] and JADE [32]. jDE is a improved version of DE by proposing a self adaptive parameter setting in differential evolution to avoid the manual parameter setting of F and CR. The parameter control technique is based on the self adaptation of two parameters associated with the evolutionary process. DE/BBO is a hybrid differential evolution algorithm, which hybridizes the DE operator with the migration of BBO. In JADE, a normal distribution and a Cauchy distribution are utilized to generate F and CR for each target vector, respectively. Table 6 summarizes the experimental results. As can be seen in this table, surprisingly, the standard DE significantly outperforms the up-to-date DE algorithms for suleymankoy anomaly, Weiss anomaly and Nalbant cesmesi anomaly. For the suleymankoy anomaly, DE can provide 28 successes rate compared with the 10, 8, 2 times of jDE, DE/BBO and JADE. For Weiss anomaly, DE can win 29 successes rate compared with the 5, 10, 3 times of jDE, DE/BBO and JADE. For Nalbant cesmesi anomaly, DE can generate better solutions than other algorithms and provide 22 successes rate.

**Table 6:** Compared with other state of the art DE algorithms.

problems	Algorithms	Successes	Mean iter.	Std. iterations	Mean func. eval.	Std func.eval.	Std RMS	Mean RMS	Min RMS
suleymankoy anomaly	DE	**28/30**	43.00	12.16	13200.00	3647.83	9.58	14.65	**6.546**
	jDE	10/30	29.20	8.00	9060.00	2399.17	4.47	33.51	26.465
	DE/BBO	8/30	63.63	36.04	19387.50	10811.8	4.10	35.53	27.692
	JADE	2/30	12.50	2.12	4050.00	636.40	1.06	39.06	38.305
Weiss anomaly	DE	**29/30**	40.43	25.60	12428.57	7679.60	8.15	14.99	**9.3594**
	jDE	5/30	11.20	4.44	3660.00	1331.54	2.48	36.35	34.167
	DE/BBO	10/30	76.40	39.80	23220	11940.0	5.65	31.91	22.635
	JADE	3/30	10.67	4.73	3500.00	1417.74	5.10	31.19	26.747
Nalbant cesmesi anomaly	DE	**22/30**	45.86	16.87	14059.09	5061.87	6.36	9.85	**8.0862**
	jDE	15/30	35.80	13.84	11040.00	4152.59	7.60	23.37	8.405
	DE/BBO	7/30	101.0	29.56	30600	8869.05	13.16	23.16	8.323
	JADE	1/30	28.00	0.00	8700.00	0.00	0.00	37.00	37.001

Though standard DE performs much better than these up-to-date DE variants do, it does not mean that standard DE is better than these algorithms. The reason may be that jDE, DE/BBO and JADE are all designed for the purpose of solving general unconstrained problems. Yet, in this paper, the self potential data problem is very specific. This also brings us an open problem: can the researchers propose some improved version of DE on solving the self potential data problem?

### Conclusions

This paper illustrated the application of differential evolution in estimating the self-potential data. The effectiveness of the proposed algorithm is demonstrated on three difficult instances including noise free data, contaminated synthetic data and some field data. The DE algorithm has the ability to find the better quality solution, and has better convergence characteristics and computational efficiency. The comparison of the results with other methods reported in the literature show the superiority of the proposed method and its potential for solving SP problem. From the results obtained, it is concluded DE algorithm is a promising technique for solving the quantitative interpretation of self potential data. In the future work, we will try to propose some improved version of DE to solve the problem.
